# Effectiveness of an Internet- and App-Based Intervention for College Students With Elevated Stress: Randomized Controlled Trial

**DOI:** 10.2196/jmir.9293

**Published:** 2018-04-23

**Authors:** Mathias Harrer, Sophia Helen Adam, Rebecca Jessica Fleischmann, Harald Baumeister, Randy Auerbach, Ronny Bruffaerts, Pim Cuijpers, Ronald C Kessler, Matthias Berking, Dirk Lehr, David Daniel Ebert

**Affiliations:** ^1^ Clinical Psychology and Psychotherapy Friedrich-Alexander-University Erlangen-Nuremberg Erlangen Germany; ^2^ Clinical Psychology and Psychotherapy University of Ulm Ulm Germany; ^3^ Department of Psychiatry Columbia University New York, NY United States; ^4^ Universitair Psychiatrisch Centrum Katholieke Universiteit Leuven Leuven Belgium; ^5^ Department of Clinical, Neuro, and Developmental Psychology Vrije Universiteit Amsterdam Amsterdam Netherlands; ^6^ Department of Health Care Policy Harvard Medical School Boston, MA United States; ^7^ Division of Online Health Trainings Innovation Incubator Leuphana University Lüneburg Germany

**Keywords:** randomized controlled trial, stress, psychological, depression, telemedicine, students, help-seeking behavior

## Abstract

**Background:**

Mental health problems are highly prevalent among college students. Most students with poor mental health, however, do not receive professional help. Internet-based self-help formats may increase the utilization of treatment.

**Objective:**

The aim of this randomized controlled trial was to evaluate the efficacy of an internet-based, app-supported stress management intervention for college students.

**Methods:**

College students (n=150) with elevated levels of stress (Perceived Stress Scale 4-item version, PSS-4 ≥8) were randomly assigned to either an internet- and mobile-based stress intervention group with feedback on demand or a waitlist control group. Self-report data were assessed at baseline, posttreatment (7 weeks), and 3-month follow-up. The primary outcome was perceived stress posttreatment (PSS-4). Secondary outcomes included mental health outcomes, modifiable risk and protective factors, and college-related outcomes. Subgroup analyses were conducted in students with clinically relevant symptoms of depression (Center for Epidemiological Studies’ Depression Scale >17).

**Results:**

A total of 106 participants (76.8%) indicated that they were first-time help-seekers, and 77.3% (intervention group: 58/75; waitlist control group: 58/75) showed clinically relevant depressive symptoms at baseline. Findings indicated significant effects of the intervention compared with the waitlist control group for stress (d=0.69; 95% CI 0.36-1.02), anxiety (d=0.76; 95% CI 0.43-1.09), depression (d=0.63; 95% CI 0.30-0.96), college-related productivity (d=0.33; 95% CI 0.01-0.65), academic work impairment (d=0.34; 95% CI 0.01-0.66), and other outcomes after 7 weeks (posttreatment). Response rates for stress symptoms were significantly higher for the intervention group (69%, 52/75) compared with the waitlist control group (35%, 26/75, *P*<.001; number needed to treat=2.89, 95% CI 2.01-5.08) at posttest (7 weeks). Effects were sustained at 3-month follow-up, and similar findings emerged in students with symptoms of depression.

**Conclusions:**

Internet- and mobile-based interventions could be an effective and cost-effective approach to reduce consequences of college-related stress and might potentially attract students with clinically relevant depression who would not otherwise seek help.

**Trial Registration:**

German Clinical Trial Register DRKS00010212; http://www.drks.de/drks_web/navigate.do? navigationId=trial.HTML&TRIAL_ID=DRKS00010212 (Archived by WebCite at http://www.webcitation.org/6w55Ewhjd)

## Introduction

### Background

Between 25% and 50% of college students meet the criteria for at least one mental health disorder in a given year [1,2]. Data suggest that mental disorders account for about half the disease burden of young adults in developed countries [3] and are associated with a range of negative consequences, including lowered academic performance [4] and college attrition [5].

Despite the availability of effective treatment [6], only 1 in 5 students with mental disorders receives minimally adequate treatment [1]. Reasons for this treatment gap include attitudinal barriers such as stigma and a preference for self-help [7].

Internet- and mobile-based interventions [8] might help to increase the utilization of psychological interventions, as they can be easily accessed, allow for high scalability, and can be provided at a low cost [9,10]. Internet-based interventions may also be suitable for college student populations [11], with research indicating that preference for help-seeking through the internet is higher among younger and well-educated individuals [12]. There is meta-analytic evidence suggesting the efficacy of internet interventions for a range of conditions and populations [6,13-16], including college students [17], with effect sizes of technology-delivered interventions ranging from standardized mean difference (SMD) of 0.42 to 0.43 for depression, 0.30 to 0.56 for anxiety, and 0.73 to 0.82 for stress [17,18]. However, the few results for internet and mobile-based interventions targeting stress in students are conflicting in terms of their effectiveness [19,20], warranting further research.

A recent meta-analysis suggests that intervention effects are considerably higher in indicated compared with general student populations [18], stressing the importance of developing suitable intervention approaches for at-risk students. Internet-based interventions which are labeled to improve stress coping skills, as opposed to focusing on reducing symptoms of mental disorders, could represent a promising way to reach such burdened individuals. In an Australian investigation among severely distressed college students, 55.7% indicated that they were quite or very likely to use an internet-delivered program to seek help [21]. A significant association between heightened stress levels and positive attitudes toward internet intervention usage has also been found in a German general population sample [22].

If proven to be effective, internet- and mobile-based approaches could provide a feasible instrument to help avert the onset of more severe stress-related mental health concerns in at-risk college students [8]. More research is therefore required to corroborate results on the effectiveness of internet- and mobile-based stress interventions and assess the potential of such interventions to reach and be effective in burdened students who already show symptoms of mental illness such as depression. Facing the deleterious effects of poor mental health on academic functioning, it is also important to assess whether such interventions may have an impact on important college-related outcomes such as academic self-efficacy and impairment [17].

### Objectives

The aim of this study is thus to evaluate the effectiveness of an internet- and mobile-based intervention targeting university students with heightened stress levels. We hypothesized the internet intervention to be more effective in reducing symptoms of stress compared with a waitlist control group (WCG). It was furthermore assumed that more students participating in the intervention compared with the WCG would show a reliable change in perceived stress outcomes and attain close to symptom-free status. The second objective of this study was to investigate the hypothesized positive effect of the intervention on further mental health outcomes, modifiable risk and protective factors, and college-related outcomes compared with the WCG. Finally, our aim was to explore intervention participants’ adherence to, and acceptance of, the intervention.

## Methods

This study was carried out as part of the WHO World Mental Health International College Student project [23]. The WHO World Mental Health International College Student project aims to obtain accurate cross-national information on the prevalence, incidence, and correlates of mental, substance, and behavioral problems among college students worldwide, to describe patterns of service use and unmet need for treatment, to investigate the associations of these disorders with academic functioning, and to evaluate the effects of a wide range of preventive and clinical interventions on student mental health, functioning, and academic performance.

### Design

A 2-armed randomized controlled trial was conducted with 150 participants, comparing an internet and app-based intervention with feedback on demand (*StudiCare Stress*) to a waitlist control group (WCG). Both conditions had full access to treatment as usual (TAU). The sample size allowed to detect effect sizes of *d*=0.41 with a power (1− *β*) of 0.80 with alpha of .05 and was based on a meta-analysis on internet-based interventions for college students, which reported an SMD of 0.73 for stress but lower effects for depression outcomes (SMD=0.43) [17]. A sample size of 150 was therefore chosen to also detect significant changes for secondary outcomes in this study such as depression.

**Figure 1:**
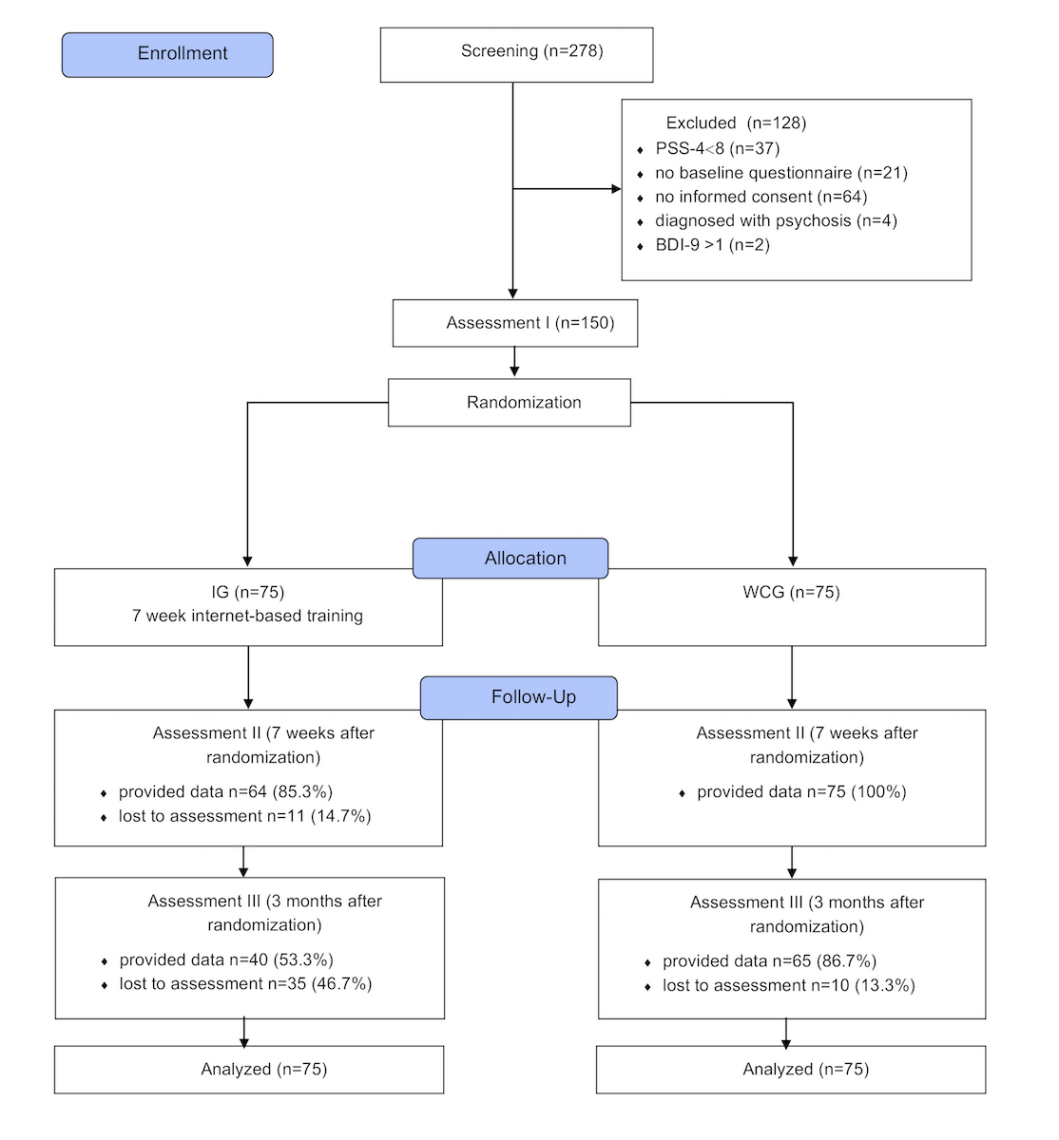
Flow of participants (CONSORT flow chart). BDI: Beck Depression Inventory; IG: intervention group; WCG: waitlist control group; CONSORT: Consolidated Standards of Reporting Trials.

Assessments took place at baseline (T1), posttreatment (T2; 7 weeks), and 3 months after baseline (T3; see Figure 1). Self-report data were collected using a Web-based assessment tool (Advanced Encryption Standard, 256-bit encryption). All procedures involved in the study were consistent with the generally accepted standards of ethical practice. The study was approved by the University of Erlangen-Nuremberg ethics committee (Erlangen, Germany; 322_15 B). The trial is registered in the German Clinical Trials Register (DRKS00010212 [24]). This study describes the main effectiveness analysis of the intervention; we also assessed moderator and mediator variables, which are listed in the trial registration (see Multimedia Appendix 1) and will be analyzed and reported in due length elsewhere.

### Participants

Inclusion criteria were (1) elevated levels of perceived stress (Perceived Stress Scale 4-item version, PSS-4≥8 [25]; representing a level of stress one *SD*=2.92 above the mean of 4.49 in a large student sample [25]), (2) enrollment in a German-speaking university at the beginning of the training, (3) age ≥18 years, (4) internet access, (5) willingness to provide self-report data at all assessment points, and (6) informed consent. Exclusion criteria were (1) self-reported diagnosis of dissociative symptoms or psychosis in the past or (2) considerable risk for suicide (Beck Depression Inventory item 9 >1; “I feel I would be better off dead” or “I would kill myself if I had the chance”). Individuals showing an elevated risk for suicide were given detailed information about treatment options and were asked to see a physician or psychiatrist as soon as possible.

### Recruitment

Participants were recruited via university press reports, student counseling services, and social media platforms. Potential participants declared interest in partaking in the study by filling out a Web-based registration form on the study website.

### Assessment of Eligibility and Randomization

Individuals who declared interest in participating received an information letter along with an informed consent sheet and were asked to provide an email address for their intervention platform profile. Applicants were informed that withdrawal from the study was possible at any time, did not go along with any negative consequences, and all collected case data could be deleted on request during the study. Interested participants were asked to complete the written informed consent form and fill out the Web-based screening questionnaire.

Individuals meeting all the inclusion and none of the exclusion criteria were invited to fill out the baseline assessment. After completion, individuals were randomly allocated to either the IG or the WCG. Randomization took place at a ratio of 1:1 and a block size of 2 using an automated computer-based random integer generator (*Randlist*, Datinf GmbH, Tübingen, Germany) and was performed by a researcher not otherwise involved in the study. Participants could not be blinded to study conditions; yet, during the randomization process, the allocation was concealed from participants, researchers involved in recruitment, and e-coaches.

### Study Conditions

#### Intervention Condition

The framework for *StudiCare Stress* was derived from *GET.ON Stress*, a Web-based stress management intervention for employees [26]. Changes in form and therapeutic content were made to tailor the intervention to university students’ needs.

The intervention is based on cognitive-behavioral and third-wave techniques and aligns with Lazarus’ transactional model of stress [27] in differentiating between problem-focused and emotion regulation–focused coping. For problem-focused coping, cognitive-behavioral problem-solving strategies are applied to reduce and eliminate modifiable stressors. Emotion regulation refers to the processes through which individuals monitor, evaluate, modify, and thus control emotions to reach relevant needs or goals and has been shown to be influential in reducing various symptoms of mental illness [28]. Elective modules integrated at the end of session 2 to 7 could be chosen based on individual need and interest, covering student-specific topics: social support, rumination and worrying, time management, procrastination, test anxiety, sleep, motivation, nutrition and exercise, and dealing with writer’s block and concentration.

The intervention comprised 8 main modules. Completing 1 module took 30 to 90 min, and participants were advised to complete at least one and a maximum of 2 modules per week. Thus, the intervention was intended to be completed in about 5 to 7 weeks (see Multimedia Appendix 2 for a detailed description of the modules).

Strong emphasis was put on the transfer of acquired knowledge, strategies, and techniques into the students’ daily life through homework assignments. A personal diary app could be downloaded by participants to keep track of mood fluctuations, monitor factors contributing to their stress levels and reflect on intervention elements they could implement into their daily life. The diary app was introduced in module 1 as an adjunct to the main sessions and contained standardized free-text fields, rating scales, and gave the opportunity to add a photo to the entry (see Textbox 1). Participants were also provided with a PDF version of the diary and were instructed to monitor their mood 2 to 3 times each week, using either the app or a printout of the PDF for their entries.

In addition, before beginning with the intervention, participants could request automatic daily messages containing short motivational prompts and ultrabrief training exercises via SMS (short message service), aimed at facilitating transfer of learned strategies into daily life routine. Messages were prescheduled to roughly mirror content and exercises provided through the progression of the intervention.

Participants were guided by an eCoach, a trained student in a master’s program in psychology. Contact between the eCoach and intervention participants was solely established online, and there were no face-to-face meetings. An adherence-focused guidance concept in accordance with the human accountability model [29,30] was applied, which has been shown to be noninferior to intensive guidance while minimizing human resources ([31]; for a detailed description see [26]). Guidance consisted of 3 parts: (1) monitoring adherence (sending up to 3 reminders when a module was not completed during 1 week through the internal platform messaging system and via email), (2) checking the intervention platform back-end for participants who had completed a new module to unlock the next module and send standardized motivational messages through the platform, and (3) providing feedback on demand. When requesting help, participants received feedback within 48 hours.

The feedback reflected the participants’ individual questions and problems and gave positive reinforcement. Feedback on demand was available for each participant from module 1 until completion of the booster session and was given via the internal messaging system of the training platform. Only few participants (5%, 4/75) requested individual feedback, resulting in 5 content feedbacks for the entire sample. In total, the eCoach sent 289 reminders (3.85 reminders per participant).

#### Control Condition

Students assigned to the waitlist control condition (WCG) completed the same assessments at T1, T2, and T3 as the intervention condition, but were not given access to the intervention until 3 months after randomization. Yet, they had full access to TAU offered by routine health care.

**General structure of the app-based diary entries.**

1. How do you feel today? (Emoticons: Happy–Sad–Anxious–Angry)
2. How stressed out do you feel today? (Rating scale 1-10)
3. Describe what happened today. (Free text)
4. Were you able to identify any things contributing to your stress levels today? (Free text)
5. Are there any techniques you previously learned that you may be able to apply? (Free text)
6. Do you want to add a photo to your entry? (Upload button)

### Primary Outcome Measure

The primary outcome was perceived stress as measured by the PSS-4 [25]. The PSS-4 assesses the degree to which individuals evaluate their lives as stressful, especially regarding how uncontrollable and overloading relevant aspects of life are perceived. The PSS-4 comprises 4 items (Item 1: “How often have you felt you were unable to control the important things in your life?”; Item 2: “How often have you felt confident about your ability to handle your personal problems?”; Item 3: “How often have you felt that things were going your way?”; Item 4: “How often have you felt difficulties were piling up so high that you could not overcome them?”), yielding a score between 0 and 16. Participants rated their level of perceived stress within the last 2 weeks on a 5-point Likert scale (0= *never*; 4= *very often*). A two-factor structure has been commonly found for the PSS [32-35], with positively framed items representing perceived coping self-efficacy and negative items reflecting hopelessness, the latter being a strong predictor for depression [36]. Higher scores on the PSS have shown to have good predictive validity for several adverse health outcomes [36-38]. Despite its brevity, the PSS-4 has been found to have acceptable to good psychometric properties [39,40]. The scale has a good level of internal consistency in this study as indicated by a Cronbach alpha of .83.

### Secondary Outcome Measures

Unless otherwise specified, all outcomes were measured for a retrospective time frame of 2 weeks. All measures were administered in German. When no German translation was available, scales were translated independently by two of the researchers (MH and SHA), who then compared and discussed the translations to resolve disagreement.

#### Mental Health

To examine effects of the stress intervention on symptoms of common mental disorders, we included mental health outcomes associated with elevated distress in college students, including depression (short German form of the Center for Epidemiological Studies’ Depression Scale, CES-D [41]; 15 items, scale 0-3, range 0-45) and state anxiety (Spielberger State-Trait Anxiety Inventory [42]; 6 items, scale 1-4, range 6-24; *at the moment*) [43]. General well-being as an overall marker of mental health was assessed by the WHO-Five Well-Being Index (WHO-5 [44]; 5 items, scale 0-6, range 0-30), and emotional exhaustion using the Maslach Burnout Inventory-student version ([45]; 5 items, scale 1-6, range 5-30).

#### Risk and Protective Factors

Following measures for established risk and protective factors were assessed to investigate the intervention’s effect on individual resources and vulnerabilities related to the development and proliferation of mental illness: dysfunctional perfectionism [46] (Revised Almost Perfect Scale [47]; translated; 8 items, scale 1-7, range 8-56), resilience [48] (Connor-Davidson Resilience Scale short form [49]; translated; 2 items, scale 0-4, range 0-8), self-compassion [50,51] (Self-Compassion Scale [52]; 12 items, scale 1-5, range 12-60), and self-esteem [53] (Rosenberg Self-Esteem Scale [54]; 10 items, scale 1-4, range 10-40).

#### College-Related Outcomes

To evaluate presenteeism and loss of productivity, the Presenteeism Scale for Students’([55]; translated) subscale for work impairment (Work Impairment Scale; 10 items, scale 1-5, range 10-50) was administered. Productivity losses were assessed by an adaption of the Presenteeism Scale for Students’ work output scale, investigating the current percentage to which participants were able to reach their usual academic productivity. Productivity could be rated on a visual analog scale ranging from 0%= *completely unproductive* to 100%= *full productivity*. Academic self-efficacy was measured by the academic self-efficacy scale (Wirkstud [56]; 7 items, scale 1-4, range 7-28), and academic worrying using the Academic Worrying Questionnaire ([57]; translated; 10 items, scale 0-4, range 0-40).

#### Additional Measures

Additional questionnaires assessed demographic variables, prior contact with professional health providers, and satisfaction with the intervention (IG only; Client Satisfaction Questionnaire, adapted to the web context, CSQ-8 [58]; 8 items, scale 1-4). Treatment credibility and expectancies were measured at baseline by the Credibility and Expectancy Questionnaire ([59]; translated; 4 items, scale 1-5, range 4-20, 2 items, 0%-100%). Participants in the IG could give feedback on each modules’ usefulness (1= *not useful at all*, 5= *very useful*), complexity (1= *very complex*, 5= *very easy*), and duration until termination (1= *less than ½ hour*, 5= *more than 1½ hours*) on a 5-point Likert or 4-point scale, respectively.

### Statistical Analyses

#### Main Effectiveness Evaluation

All results are reported according to the Consolidated Standards of Reporting Trials statement ([60]; see Multimedia Appendix 3). Analyses based on the intention-to-treat (ITT) principle were conducted, with missing data imputed using a Markov chain Monte Carlo multivariate imputation algorithm (multiple imputation functions in IBM SPSS 23; IBM Corp, Armonk, NY, USA) with 100 estimations per missing and all variables set as predictors for imputation. Imputed datasets were then aggregated to obtain 1 imputed dataset.

The hypothesized superiority of the internet intervention was tested with regard to (1) change in participants’ perceived stress and secondary outcomes from baseline (T1) to post intervention (T2) and 3-month follow-up (T3), (2) the number of participants with treatment response, (3) the number of students achieving close to symptom-free status, and (4) the amount of participants who experienced symptom deterioration.

Differences in change of perceived stress between study arms were assessed using univariate analysis of covariance (ANCOVA) with scores at baseline as covariate to control for varying degrees of baseline scores. Effect sizes (Cohen's *d*) were calculated based on the imputed dataset for between-group differences, using the pooled IG and WCG SD [61]. To calculate 95% CIs, the formula by Rosnow and Rosenthal [62] was used. According to Cohen [63], *d*=0.2 can be considered a small effect, *d*=0.5 a medium and *d*=0.8 a large effect. A significance level of .05 (2-sided) was used for all analyses.

To ascertain the number of participants attaining a reliable improvement in stress symptomatology, participants were coded as responders or nonresponders according to the Reliable Change Index [64]. Accordingly, response was attained when participants’ scores on the PSS-4 differed more than −2.17 points from baseline to T2 and T3, respectively. Furthermore, the numbers needed to treat (NNT) to achieve 1 additional treatment response were calculated. Negative effects of the intervention were evaluated by the number of participants with reliable symptom deterioration concerning perceived stress through the Reliable Change Index. Participants were defined as symptom-free when scoring more than 2 SDs below the mean at baseline for the full sample (PSS-4 ≤7.29).

#### Subgroup Analysis

To estimate the interventions’ efficacy in a clinical population, a subgroup analysis was conducted including only participants with a score of >17 on the CES-D short form at baseline and following the same procedure as the main analysis. A score of 18 has been shown to be a valid cut-off in indicating a high probability of clinical depression [65]. Participants were classified as responders if they showed reliable change in depressive symptoms according to the Reliable Change Index.

#### Study Completer Analysis

Completer analysis based on the sample of participants who provided data at all 3 assessment points was conducted additionally as a sensitivity analysis.

### Process Evaluation

Descriptive statistics were used for process evaluation. To assess overall user satisfaction across various domains, item data provided by the CSQ-8 was examined individually. Acceptance of intervention modules was analyzed using the module feedback of the IG. Adherence was assessed by analyzing intervention completion rates tracked within the intervention platform. Finally, we analyzed the proportion of participants who accessed the diary app and requested automated short messages via SMS.

## Results

Recruitment for the study started on May 9, 2016. The last follow-ups were completed on January 30, 2017.

### Participants

The study flow can be found in Figure 1. Participants who were lost to follow-up at T2, T3, or both assessments did not differ significantly from participants who adhered to the protocol on any baseline characteristic (all *P*>.05). Table 1 summarizes detailed baseline characteristics of study participants. The majority (76.8%, 106/138) of the participants indicated that they had not consulted a physician, psychotherapist, or counselor for their health-related problems and may thus be considered first-time help-seekers. Descriptive data including all 3 assessment points for all outcomes is depicted in Table 2. Both study arms did not differ significantly (all *P*>.05) on any characteristic at baseline.

### Main Effectiveness Analysis

#### Changes in Perceived Stress

As hypothesized, the ANCOVA controlling for baseline scores revealed a significant effect for perceived stress at posttest (*F*_1,147_=19.70, *P*<.001) and at 3-month follow-up (*F*_1,147_=15.10, *P*<.001; see Table 3), with moderate to large effect sizes at both T2 (*d*=0.69; 95% CI 0.36-1.02) and T3 (*d*=0.57; 95% CI 0.24-0.89).

#### Treatment Response for Perceived Stress

Chi-squared tests revealed that significantly more participants in the IG (69%, 52/75) were classified as responders compared with the WCG (35%, 26/75) at posttest (χ^2^_1_=18.1, *P*<.001), resulting in an NNT of 2.89 (95% CI 2.01-5.08). At 3-month follow-up, 55 of 75 participants in the IG (73%) and 33 of 75 in the WCG (44%) were coded as responders (χ^2^_1_=13.3, *P*<.001) which equals an NNT of 3.41 (95% CI 2.25-7.00).

#### Symptom-Free Status for Perceived Stress

Symptom-free status was achieved by significantly (χ^2^_1_=6.7, *P*=.01) more participants in the IG ( 44%, 33/75) compared with the WCG (24%, 18/75) at T2, and at T3 (IG: 53%, 40/75; WCG: 35%, 26/75) with χ^2^_1_=5.3 (*P*=.02), resulting in an NNT of 5 (T2; 95% CI 2.87-19.31) and 5.36 (T3; 95% CI 2.92-32.66), respectively.

#### Symptom Deterioration for Perceived Stress

Only a small proportion of participants experienced symptom deterioration. Fewer participants’ stress symptomatology deteriorated in the IG (0%, 0/75) compared with the WCG, where 7 of 75 (9%) participants’ symptoms deteriorated (χ^2^_1_=7.3, *P*<.001; NNT=10.58, 95% CI 6.19-44.18) at T2. Symptom deterioration did not differ at T3, with 1 case of 75 participants (1%) in the IG and 3 of 75 (4%) in the WCG (χ^2^_1_=1.0, *P*=.31).

**Table 1:** Baseline characteristics.

Characteristics		All participants (N=150)	Intervention (N=75)	Control (N=75)
**Sociodemographics**				
	Age in years, mean (SD)	24.1 (4.1)	24.0 (4.6)	24.2 (3.6)
	Gender, female, n (%)	112 (74.7)	54 (72)	58 (77)
	In a relationship, n (%)	79 (52.7)	39 (52)	40 (53)
	Married, n (%)	6 (4.0)	4 (5)	2 (3)
**Major**				
	Business & Economics, n (%)	33 (22.0)	16 (21)	17 (23)
	Computer Science & Engineering, n (%)	13 (8.7)	9 (12)	4 (5)
	Education, n (%)	17 (11.3)	7 (9)	10 (13)
	Humanities, n (%)	12 (8.0)	5 (8)	7 (9)
	Law, n (%)	6 (3.3)	2 (3)	4 (5)
	Medicine, n (%)	15 (10.0)	7 (9)	8 (11)
	Natural Sciences, n (%)	20 (13.3)	11 (15)	9 (12)
	Social Sciences, n (%)	34 (22.7)	18 (24)	16 (21)
	Number of semesters (previous studies included), mean (SD)	6.7 (3.6)	6.4 (3)	7.07 (3.9)
**Type of tertiary education facility, n (%)**				
	College	119 (79.3)	56 (74)	63 (84)
	University of Applied Sciences	31 (20.6)	19 (25)	12 (16)
**Housing situation, n (%)**				
	Alone	31 (20.7)	18 (24)	13 (17)
	Flat share	95 (63.3)	48 (64)	47 (63)
	With parents	24 (16.0)	9 (12)	15 (20)
**Main source of funding, n (%)**				
	Parents	64 (42.7)	33 (44)	31 (41)
	Job	48 (32.02)	25 (33)	23 (31)
	Loan	34 (22.7)	15 (20)	19 (25)
	Partner	2 (1.3)	1 (1)	1 (1)
	Scholarship	2 (1.3)	1 (1)	1 (1)

### Secondary Outcome Analysis

Table 3 summarizes the results of the ITT analyses for the secondary outcomes. ANCOVAs revealed significant effects (*P*<.05) in favor of the IG for the majority of outcomes at both assessment points, with effect sizes ranging from *d*=0.33 (95% CI 0.01-0.65) for productivity (T2) to *d*=0.82 (95% CI 0.49-1.15) for emotional exhaustion (T2). No statistically significant effect was found for perfectionism (*F*_1,147_=0.38, *P*=.53) at T2, but at T3 (*P*<.001). Resilience (T2: *F*_1,147_=1.69, *P*=.17; T3: *F*_1,147_=2.94, *P*=.08), self-compassion (T2: *F*_1,147_=2.97, *P*=.09; T3: *F*_1,147_=1.46, *P*=.23), and self-esteem (T2: *F*_1,147_=0.15, *P*=.70; T3: *F*_1,147_=1.36, *P*=.25) did not differ significantly between both study arms at both assessment points.

### Subgroup Analysis

More than three-fourths of the participants (77.3%; IG: 58/75; WCG: 58/75) showed symptoms above the cut-off for clinically relevant symptoms of depression at baseline. Between-group effect sizes for depression in this subgroup were moderate to large, both for T2 (*d*=0.67, 95% CI 0.34-1.00) and T3 (*d*=0.73, 95% CI 0.40-1.06). Treatment response was achieved by 36 (62%; T2) and 33 of 58 participants (57%, T3) in the IG compared with 14 of 58 participants (24%; T2 and T3) in the WCG, resulting in an NNT to achieve one additional treatment response in the IG compared with the WCG of 2.64 (95% CI 1.83-4.70) for T2 (χ^2^_1_=17.0, *P*<.001) and 3.05 for T3 (95% CI 2.02-6.28, χ^2^_1_=12.9, *P*<.001).

**Table 2:** Means and SDs of the intervention group (intervention) and waitlist control group (control) for the intention-to-treat-sample at baseline, posttest (7 weeks), and 3-month follow-up.

Outcome and assessment point			Intervention (N=75)	Control (N=75)
			Mean (SD)	Mean (SD)
**Primary outcome**				
	**Perceived stress (low to high 0-16)**			
		Baseline	11.13 (1.93)	11.03 (1.87)
		7 weeks	7.43 (2.93)	9.49 (3.06)
		3 months	6.96 (2.73)	8.66 (3.26)
**Mental health**				
	**Depression (0-45)**			
		Baseline	24.31 (9.06)	23.97 (8.63)
		7 weeks	15.88 (8.85)	21.47 (8.96)
		3 months	16.79 (8.72)	21.92 (9.53)
	**Anxiety (6-24)**			
		Baseline	16.05 (3.37)	15.77 (4.22)
		7 weeks	13.37 (3.51)	16.03 (3.48)
		3 months	13.33 (3.59)	15.50 (4.10)
	**Well-being (0-30)^a^**			
		Baseline	8.01 (4.34)	8.81 (3.69)
		7 weeks	11.93 (5.03)	9.36 (4.35)
		3 months	12.62 (5.34)	10.57 (4.81)
	**Emotional exhaustion (5-30)**			
		Baseline	21.63 (4.49)	22.27 (4.31)
		7 weeks	18.43 (5.64)	22.36 (3.77)
		3 months	20.04 (5.08)	22.30 (4.45)
**Risk and protective factors**					
	**Dysfunctional perfectionism (8-56)**			
		Baseline	44.29 (7.90)	43.89 (7.50)
		7 weeks	43.02 (7.22)	43.45 (7.34)
		3 months	41.05 (5.94)	44.33 (6.67)
	**Resilience (0-8)^a^**			
		Baseline	4.80 (1.72)	4.79 (1.87)
		7 weeks	5.38 (1.85)	5.05 (1.97)
		3 months	5.56 (1.36)	5.17 (1.61)
	**Self-compassion (12-60)^a^**			
		Baseline	33.95 (3.47)	34.54 (3.23)
		7 weeks	34.95 (5.67)	34.16 (3.72)
		3 months	35.25 (3.26)	34.78 (3.96)
	**Self-esteem (10-40)^a^**			
		Baseline	29.25 (2.58)	29.20 (2.78)
		7 weeks	29.14 (3.61)	28.93 (2.61)
		3 months	30.10 (3.03)	29.54 (2.68)
**College-related outcomes**					
	**Academic work impairment (10-50)**			
		Baseline	28.29 (5.34)	27.88 (5.39)
		7 weeks	25.54 (5.83)	27.55 (6.13)
		3 months	24.74 (5.06)	27.44 (6.22)
	**Academic productivity (percent)^a^**			
		Baseline	52.79 (27.04)	54.30 (23.03)
		7 weeks	60.36 (24.12)	52.36 (24.16)
		3 months	67.76 (17.27)	58.21 (23.62)
	**Academic self-efficacy (7-28)^a^**			
		Baseline	17.04 (4.46)	16.34 (4.04)
		7 weeks	18.35 (4.03)	16.43 (4.12)
		3 months	18.60 (3.86)	16.37 (4.12)
	**Academic worrying (0-40)**			
		Baseline	22.35 (6.63)	22.01 (6.01)
		7 weeks	18.29 (6.16)	21.71 (5.94)
		3 months	17.82 (6.97)	21.14 (6.18)
**Treatment expectancies (0-100)**				
		Baseline	62.34 (13.51)	62.44 (16.03)

^a^Higher scores indicate better outcomes.

### Completer Analysis

The results of the completer analyses were similar to the ITT analyses, with moderate to large between-group effect sizes for the primary outcome at T2 (IG: mean=6.72, SD 2.86; WCG: mean=9.32, SD 3.16; *F*_1,88_=18.60, *P*<.001; *d*=0.85, 95% CI 0.44-1.27) and T3 (IG: mean=6.41, SD 2.84; WCG: mean=8.65, SD 3.43; *F*_1,88_=13.41, *P*<.001; *d*=0.69, 95% CI 0.29-1.10). In contrast to the main analysis, however, resilience had increased significantly in the IG compared with the WCG at T2 (*F*_1,88_=8.56, *P*=.004; *d*=0.46, 95% CI 0.06-0.86).

### Process Evaluation

#### Adherence to the Intervention

On average, participants in the IG completed 5.05 modules (SD 2.78), which equals 72.1% of the intervention. Participants completed optional add-on modules in the majority (82.1%) of sessions in which they were available. Most participants completed rumination & worrying (59%, 44/75), whereas only 8 of the 75 participants completed social support (11%). In all, 46 of the 75 participants in the IG (61%) downloaded and logged into the diary app at least once. Activation of the automated SMS messages was requested by 4 of 75 participants in the IG (5%) during the study.

#### Client Satisfaction

Overall client satisfaction with the intervention was high (see Table 4).

#### Perceived Usefulness, Difficulty, and Duration of Sessions

Most participants described the 8 treatment modules as useful and not overly complex (see Multimedia Appendix 4). Reported session duration was high, with participants having spent the most time on module 6 (Self-compassion; 28% spending more than 1 hour 30 min, 10/36).

**Table 3:** Results for the intention-to-treat sample for analyses of covariance for between-group effects, effect sizes (Cohen's *d*) for primary and secondary outcomes at posttest (7 weeks; T2) and 3-month follow-up (T3).

Outcome and assessment point			Effect size		ANCOVA^a^	
			Cohen's *d*	95% CI	*F*_1147_	*P* value
**Primary outcome**							
	**Perceived stress**					
		7 weeks	0.69	0.36 to 1.02	19.70	<.001
		3 months	0.57	0.24 to 0.89	15.10	<.001
**Mental health**							
	**Depression**					
		7 weeks	0.63	0.30 to 0.96	22.31	<.001
		3 months	0.56	0.24 to 0.89	16.62	<.001
	**Anxiety**					
		7 weeks	0.76	0.43 to 1.09	28.20	<.001
		3 months	0.56	0.24 to 0.89	14.68	<.001
	**Well-being**					
		7 weeks	0.55	0.22 to 0.87	21.06	<.001
		3 months	0.40	0.08 to 0.73	12.14	.001
	**Emotional exhaustion**					
		7 weeks	0.82	0.49 to 1.15	30.67	<.001
		3 months	0.59	0.26 to 0.92	8.93	.003
**Risk and protective factors**							
	**Dysfunctional perfectionism**					
		7 weeks	0.06	−0.26 to 0.38	0.38	.54
		3 months	0.52	0.19 to 0.84	15.79	<.001
	**Resilience**					
		7 weeks	0.17	−0.15 to 0.49	1.69	.17
		3 months	0.26	−0.06 to 0.58	2.94	.08
	**Self-compassion**					
		7 weeks	0.17	−0.16 to 0.49	2.97	.09
		3 months	0.13	−0.19 to 0.45	1.46	.23
	**Self-esteem**					
		7 weeks	0.07	−0.25 to 0.39	0.15	.70
		3 months	0.19	−0.13 to 0.51	1.36	.25
**College-related outcomes**							
	**Academic work impairment**					
		7 weeks	0.34	0.01 to 0.66	6.57	.01
		3 months	0.48	0.15 to 0.80	10.57	.001
	**Academic productivity**					
		7 weeks	0.33	0.01 to 0.65	4.29	.04
		3 months	0.46	0.14 to 0.79	9.68	.002
	**Academic self-efficacy**					
		7 weeks	0.49	0.16 to 0.81	12.74	<.001
		3 months	0.56	0.23 to 0.88	17.98	<.001
	**Academic worrying**					
		7 weeks	0.56	0.24 to 0.89	27.41	<.001
		3 months	0.50	0.18 to 0.83	16.04	<.001

^a^ANCOVA: analysis of covariance.

**Table 4:** Clients’ satisfaction with the intervention (T2; Intervention Group only).

Ratings	n (%)
Quality of the training rated as excellent or good	59 (92)
Indication that the training was the kind of intervention they wanted to receive (generally or definitely)	51 (80)
Indication that the own needs were almost all or mostly met	47 (73)
Inclination to recommend the training to a friend in need of similar help	58 (91)
Satisfaction with the amount of help received (mostly or very satisfied)	51 (80)
Indication that the training has helped (a great deal) to deal more effectively with problems	53 (83)
Satisfaction with the training in a general, overall sense (mostly or very satisfied)	55 (86)
Inclination to use the training again if in need for help	49 (77)

## Discussion

### Principal Findings

Results of this study indicate moderate to large intergroup effects for the reduction of perceived stress and other relevant health- and college-related outcomes, as well as substantial effects in individuals with clinically relevant symptoms of depression, which were highly prevalent in our recruited sample. No significant effects were found for self-compassion, perfectionism (T2), resilience, and self-esteem.

The benefits of this intervention were larger than those found in previous trials evaluating internet-based stress interventions in college students [19,20], albeit somewhat smaller than the reported overall effect of technology-delivered skill training interventions on perceived stress [18], and comparable to internet-based stress interventions in general, as reported in a recent meta-analysis, with a pooled standardized mean difference of *d*=0.64 (95% CI 0.50-0.79; perceived stress) in guided internet- and mobile-based interventions [66]. The study further contributes to current literature by showing that targeting perceived stress in students does not only result in better mental health–related outcomes and well-being but can also have a substantial beneficial impact on college-related outcomes which, to the best of our knowledge, have not been investigated so far.

Participants’ adherence to the intervention was satisfying, and the intervention was well accepted among the large majority of students. Participant feedback on the length of specific modules, however, suggests that participants may have spent more time than anticipated on some of the modules. This may indicate that some modules could be shortened to attempt to further improve adherence. Whether shortening might, in fact, result in higher adherence to the intervention, however, is not fully clear. Earlier research has reported higher adherence rates for shorter interventions, albeit focusing on the number of modules used [67]. Whether this effect also holds true for the length of specific modules remains unknown. Shortening some of the modules might potentially optimize adherence but may also compromise the intervention’s overall efficacy due to less potentially beneficial information or techniques being conveyed and trained. It has been argued that various ways in which participants prioritize provided content may lead to positive outcomes, and the ability to progress through interventions at one’s own pace might represent a key asset of internet-delivered treatment [68]. Qualitative interviews conducted with participants of this intervention suggest that the elective mini-modules for various student-relevant topics were very well accepted [69]. Providing larger amounts of content in a flexible way, allowing participants to tailor the intervention to their specific needs, could, therefore, be a promising approach to optimize intervention usage patterns [70]. However, research is warranted to test whether this might further increase adherence.

### Limitations

This study has some limitations. First, women were overrepresented in the study sample, as frequently seen in preventive interventions. Second, study dropout in the IG at 3-month follow-up was relatively high and larger in the IG. Albeit being a common limitation in clinical trial research [71], and with differential dropout rates having been reported before for internet-based stress intervention trials in tertiary education students [72,73], this restricts the generalizability of our findings on long-term effects. Attrition analysis, however, did not result in any significant baseline differences between dropout and nondropout cases, which may be an indicator that results were not overly biased due to unequal dropout [74]. Third, because of ethical reasons, participants had full access to treatment-as-usual. Thus, we cannot rule out potential cointervention effects due to utilization of health services. Finally, because of feasibility and ethical reasons, such as not denying one half of the sample access to the intervention they sought after, participants in the IG of this study were compared with a WCG to assess effects of the training. The influence of treatment and change expectancies have been discussed as an artifact in clinical evaluation trials using WCGs because they potentially discourage participants with delayed access to treatment to initiate health-related behavior changes, and thus lead to accentuate effects [75].

Most college students with depression do not seek treatment through conventional health care channels [76], and attitudinal barriers, such as fear of stigmatization, have been shown to have a large impact on treatment utilization [77]. As our findings indicate that (1) a large number of students in this sample did not use conventional treatment options before, (2) the majority of students who were willing to use this intervention reported clinically significant symptoms of depression, and (3) among this group, the treatment response was favorable; future studies should explore whether internet-delivered stress interventions, labeled as providing “support for coping with academic stress,” might potentially attract students with symptoms of depression who would not use formal mental health treatment and whether they can reduce the incidence of depressive disorders [8]. Future studies should thus investigate the utility of internet and mobile-based interventions in affected students, that is, with symptoms of major depression, as an indicated preventative or early intervention approach to narrow the treatment gap and improve academic functioning [4].

### Conclusions

In conclusion, internet- and mobile-based interventions could be an acceptable, effective, and potentially cost-effective approach to reduce the negative consequences associated with college-related stress.

## Appendix

Multimedia Appendix 1 Trial registration.

Multimedia Appendix 2 Intervention modules.

Multimedia Appendix 3 CONSORT-EHEALTH checklist (V 1.6.1).

Multimedia Appendix 4 Participants’ perceived usefulness, complexity and duration for each intervention module.

## Abbreviations

ANCOVA: analysis of covariance
CES-D: Center for Epidemiological Studies’ Depression Scale
IG: intervention group
ITT: intention-to-treat
NNT: numbers needed to treat
PSS-4: Perceived Stress Scale, 4-item version
SMD: standardized mean difference
SMS: short message service
T1: baseline assessment
T2: postassessment (7 weeks)
T3: 3-month follow-up assessment
TAU: treatment as usual
WCG: waitlist control group
