# Supplementary material for: Effectiveness of an Internet- and App-Based Intervention for College Students With Elevated Stress: Randomized Controlled Trial
Source: J Med Internet Res. 2018 Apr 23;20(4):e136. doi: 10.2196/jmir.9293 (PMC5938594; doi:10.2196/jmir.9293)
Supplement: Multimedia Appendix 1 [file jmir_v20i4e136_app1.pdf]

Trial Description

Title

Online based self-help stress management program for students with feedback on demand

Trial Acronym

StudiCare

URL of the trial

http://www.studicare.de

Brief Summary in Lay Language

Within the last few years, accumulated stress factors among students increased due to the restructuring of the study system of the Bologna process. These stress factors can be harmful for body and well-being. The amount of diagnosed mental illnesses and the subjective perception of stress accelerated as well. Therefore, there is an urgent need for appropriate measures for stress management that meet students' requirements.
StudiCare aims to reduce perceived stress and strain. It targets students who are seeking help in order to cope with personal and academic problems and to deal with difficult emotions. The aim of the study is to evaluate the efficacy of the one based, guided self-help concept compared to a waitlist control group. Participants receive feedback on demand from an online coach.

Brief Summary in Scientific Language

Stress at university is associated with a variety of mental and emotional problems, such as reduced work performance, exhaustion or anxiety. Self-help interventions offer an easier access to the student target group than face-to-face interventions. Especially online and internet based self-help interventions are the method of choice for stress students that tend to avoid formal therapeutical help. The aim of the study is to assess the efficacy and possible moderators and mediators of an internet-based stress management program for students. Within the framework of an prospective, randomized control study students (N=150) experiencing a high levels of stress will be randomly allocated into either the intervention group which gains access to the seven session intervention immediately or a waitlist control group which receives the intervention 3 months later. Participants will train on a weekly basis. After the 3 month follow-up, the participants of the waitlist control group also gets access to the intervention. The intervention is based on problem- and emotion-focused stress management according to Lazarus and contains systematic problem solving as well as effective emotion regulation strategies and practices concerning acceptance and tolerance, relaxation exercise, effective self-support and mindfulness. Participants will receive feedback on demand from an online coach and will be supported through mobile components in their daily life. Data are collected at baseline, at seven weeks and three months post randomization. Perceived stress is measured as a primary outcome. Secondary outcomes include symptoms of depression, anxiety, self-compassion, maladaptive perfectionism, presenteeism and worrying/rumination.

Stress at university is associated with a variety of mental and emotional problems, such as reduced work performance, exhaustion or anxiety. Self-help interventions offer an easier access to the student target group than face-to-face interventions. Especially online and internet based self-help interventions are the method of choice for stress students that tend to avoid formal therapeutical help. The aim of the study is to assess the efficacy and possible moderators and mediators of an internet-based stress management program for students. Within the framework of an prospective, randomized control study students (N=150) experiencing a high levels of stress will be randomly allocated into either the intervention group which gains access to the seven session intervention immediately or a waitlist control group which receives the intervention 3 months later. Participants will train on a weekly basis. After the 3 month follow-up, the participants of the waitlist control group also gets access to the intervention. The intervention is based on problem- and emotion-focused stress management according to Lazarus and contains systematic problem solving as well as effective emotion regulation strategies and practices concerning acceptance and tolerance, relaxation exercise, effective self-support and mindfulness. Participants will receive feedback on demand from an online coach and will be supported through mobile components in their daily life. Data are collected at baseline, at seven weeks and three months post randomization. Perceived stress is measured as a primary outcome. Secondary outcomes include symptoms of depression, anxiety, self-compassion, maladaptive perfectionism, presenteeism and worrying/rumination.

Organizational Data

- DRKS-ID: **DRKS00010212**
- Date of Registration in DRKS: **2016/05/12**
- Date of Registration in Partner Registry or other Primary Registry: **[---]***
- Investigator Sponsored/Initiated Trial (IST/IIT): **yes**
- Ethics Approval/Approval of the Ethics Committee: **Approved**
- (leading) Ethics Committee Nr.: **322_15B , Ethik-Kommission der Friedrich-Alexander-Universität Erlangen-Nürnberg**

Secondary IDs

Health condition or Problem studied

- ICD10: **Z73 - Problems related to life-management difficulty**
- ICD10: **F32 - Depressive episode**
- Free text: **Stress**

Interventions/Observational Groups

- Arm 1: **Intervention group: Online training consisting of 7 sessions within a time period of 7 weeks. Each session will be completed in approximately 45-60 minutes. Participants will train on a weekly basis. Participants receive feedback on demand via e-mail from an online coach. Four weeks after finishing the training, participants complete an additional booster session. Questionnaires are asked to be completed at pre- and posttest (7 weeks), as well as at 3 months follow up.**
- Arm 2: **Waitlist control group: Participants obtain access to the same training as participants of the intervention group after a waiting time of 3 months. Questionnaires are asked to be completed at pre- and posttest as well as at 3 months follow up.**

Characteristics

- Study Type: **Interventional**
- Study Type Non-Interventional: **[---]***
- Allocation: **Randomized controlled trial**
- Blinding: **[---]***
- Who is blinded: **[---]***
- Control: **Control group receives no treatment**
- Purpose: **Treatment**
- Assignment: **Parallel**
- Phase: **N/A**
- Off-label use (Zulassungsüberschreitende Anwendung eines Arzneimittels): **N/A**

Primary Outcome

Perceived Stress, measured with the Perceived Stress Scale (PSS-10, Cohen, Kamarck & Mermelstein, 1983) at Baseline, after the training (7 weeks) and at 3-months follow-up.

Secondary Outcome

- suicide risk (Beck Depressions Inventar, Hautzinger, Keller & Kühner, 2006): pre

- Conscientiousness (Facets of Conscientiousness, MacCann, Duckworth, Roberts, 2008): pre

- credibility and expectancy of the training (credibility/expectancy questionnaire, Devilly & Borkovec, 2000): pre

- Willingness to pay (Willingness to pay questionnaire, Keith, Haddon, Birch, 2000): pre

- depressive symptoms (allgemeine Depressionsskala, Hautzinger & Bailer1993): pre, post (7 weeks), 3- month-Follow-Up

- subjective perceived stress and emotional exhaustion (Maslach Burnout Inventory für Studenten, Hamborg, Schoppe, Braun & Straatmann, 2012): pre, post (7 weeks), 3- month-Follow-Up

- subjective perceived quality of life (WHO 5- Wohlbefindens-Index, Brähler, Mühlan, Albani & Schmidt, 1998): pre, post (7 weeks), 3- month-Follow-Up

- Concerns towards university life: (Academic worry questionnaire, Wolitzky & Telch, 2005): pre, post (7 weeks), 3- month-Follow-Up

- Perfectionism (Almost Perfect Scale Revised, Slaney, Rice, Mobley, Trippi, Ashby, 2001): pre, post (7 weeks), 3- month-Follow-Up

- reduced ability to work due to health problems (Presenteeism Scale for Students, Matsushita et al., 2011): pre, post (7 weeks), 3- month-Follow-Up

- risk and protective factors (Rosenberg Self-Esteem Scale, Ferring & Filipp, 2003): pre, post (7 weeks), 3- month-Follow-Up

- risk and protective factors (Connor & Davidson resilience scale, Connor & Davidson, 2003): pre, post (7 weeks), 3- month-Follow-Up

- academic self-efficacy (College Self-Efficacy Inventory, Solberg, O'Brien, Villareal, Kennel & Davis, 1993): pre, post (7 weeks), 3- month-Follow-Up

- emotionregulation competence (Fragebogens zur Selbsteinschätzung Emotionaler Kompetenzen, Berking & Znoj, 2008): pre, post (7 weeks), 3- month-Follow-Up

- Amount of self-compassion (Self- Compassion Scale, Hupfeld & Ruffieux, 2011): pre, post (7 weeks), 3- month-Follow-Up

- internal/external locus of control (Multidimensional Locus of Control Scale Form C, Wallston, Stein, Smith, 1994): pre, post (7 weeks), 3- month-Follow-Up

- Repetitive negative thoughts (Behavioral Activation for Depression Scale, Fuhr et al, noch unveröffentlicht):pre, post (7 weeks), 3- month-Follow-Up

- Psychological flexibility (Acceptance and Action Questionnaire II, Lloyd et al. 2013): pre, post (7 weeks), 3- month-Follow-Up

- problem solving (Diagnostisches Inventar Problemlösen, Dirksmeier, 1991): pre, post (7 weeks), 3- month-Follow-Up

Countries of recruitment

- DE **Germany**
- CH **Switzerland**
-

AT **Austria**

Locations of Recruitment

- other **Teilnehmer werden über die Forschungswebsite studicare.de rekrutiert / participants are recruited via the research page studicare.de**

Recruitment

- Planned/Actual: **Actual**
- (Anticipated or Actual) Date of First Enrollment: **2016/05/09**
- Target Sample Size: **150**
- Monocenter/Multicenter trial: **Multicenter trial**
- National/International: **International**

Inclusion Criteria

- Gender: **Both, male and female**
- Minimum Age: **18 Years**
- Maximum Age: **no maximum age**

Additional Inclusion Criteria

Enrollment at a university or higher education institution, distinct level of perceived university related stress, internet access, valid e-mail address

Exclusion criteria

At Baseline slightly suicidal (BDI II item 9 >1), not willing to sign informed consent, diagnosed psychosis or dissociative symptoms in the past

Addresses

- **Primary Sponsor**

Friedrich-Alexander Universität Erlangen-Nürnberg, Lehrstuhl für Klinische Psychologie und Psychotherapie
*** ***
Nägelsbachstraße 25a
91052 Erlangen
Germany

Telephone: ***

Primary Sponsor

Friedrich-Alexander Universität Erlangen-Nürnberg, Lehrstuhl für Klinische Psychologie und Psychotherapie
*** ***
Nägelsbachstraße 25a
91052 Erlangen
Germany

Telephone: *****
Fax: **[---]***
E-mail:
72457531336d724c744d794a41765677536b4f766a66685650724576353 76a2b753156626253516d655a593d at drks.de
URL: **http://www.psychologie.phil.uni-erlangen.de**

- **Contact for Scientific Queries**

Friedrich-Alexander Universität Erlangen-Nürnberg, Lehrstuhl für Klinische Psychologie und Psychotherapie
*** ***
Nägelsbachstraße 25a
91052 Erlangen
Germany

Telephone: *****
Fax: **[---]***
E-mail:
72457531336d724c744d794a41765677536b4f766a66685650724576353 76a2b753156626253516d655a593d at drks.de
URL: **http://www.psychologie.phil.uni-erlangen.de**

- **Contact for Public Queries**

Friedrich-Alexander Univerität Erlangen Nürnberg
*** ***
Nägelsbachstraße 25 a
91052 Erlangen
Germany

Telephone: *****
Fax: **[---]***
E-mail:
4c70715a6255326c436b6b5946765433367658304c7667552f4f7432796877445 54870516e634433324e6f3d at drks.de
URL: **studicare.info**

Sources of Monetary or Material Support

- **Institutional budget, no external funding (budget of sponsor/PI)**

Lehrstuhl für klinische Psychologie und Psychotherapie (Klips), Institut für Psychologie

Institutional budget, no external funding (budget of sponsor/PI)

Lehrstuhl für klinische Psychologie und Psychotherapie (Klips), Institut für Psychologie
Nägelsbachstr. 25a
91052 Erlangen
Germany

Telephone: *****
Fax: **[---]***
E-mail: **[---]***
URL: **[---]***

Status

- Recruitment Status: **Recruiting complete, follow-up complete**
- Study Closing (LPLV): **2017/01/30**

Trial Publications, Results and other documents

** This entry means the parameter is not applicable or has not been set.*
**** This entry means that data is not displayed due to insufficient data privacy clearing.*
