# Supplementary material for: Effectiveness of an Internet- and App-Based Intervention for College Students With Elevated Stress: Randomized Controlled Trial
Source: J Med Internet Res. 2018 Apr 23;20(4):e136. doi: 10.2196/jmir.9293 (PMC5938594; doi:10.2196/jmir.9293)
Supplement: Multimedia Appendix 2 [file jmir_v20i4e136_app2.pdf]

Intervention Modules

Session	Name	Content	
1	*Introduction*	Psychoeducation, information about stress and preview of subsequent sessions	
2	*Problem Solving*	Stress management strategies, systematic problem solving using a 6-step individualized problem solving heuristic	
3	*Muscle- and Breath Relaxation*	Recap and modification of the problem solving heuristic, information on basic principles of muscle and breath relaxation, audio exercises for daily usage	
4	*Mindfulness*	Recap of muscle- and breath relaxation and addition of *detached mindfulness* components into the routine, metacognitive strategies for dealing with self-criticism	
5	*Acceptance and Tolerance*	Recap of metacognitive strategies, dealing with unsolvable problems, psychoeducation on and exercises for acceptance and tolerance of unpleasant emotions	
6	*Self-Compassion*	Fostering self-compassion in precarious situations, defusion of self-worth and performance, writing a self-compassionate letter, cognitive restructuring to overcome dysfunctional perfectionistic thought-action patterns	
7	*My Master Plan*	Recognizing physical warning signs, recap of coping strategies for solvable and unsolvable stressors, creating a plan for the future	
8	*Booster Session*	Further information on self-help and psychotherapy, evaluation of training transfer, recap of all sessions, repetition of previous exercises, finding future directions for development	
2-7	*Elective Modules*	*Social support*	Communication styles, receiving and providing support
		Rumination and worrying	Reflection on positive and negative aspects of worry, coping with uncertainty
		Time management	Effective time scheduling, common planning fallacies, learning to prioritize
		procrastination	Identifying situations in which procrastination occurs, strategies to reduce procrastination
		Test anxiety	Effective studying techniques, using paradoxical intentions, de-catastrophizing blackouts
		Sleep	Sleep restriction
		Motivation	Finding reasons for lacking motivation, exercising delay of gratification
		Nutrition and exercise	Creating an individual eating and exercise schedule, dealing with relapses
		Dealing with writer's block	Reasons and coping strategies for writer's block
		Concentration	Audio-based concentration exercises
