# Supplementary material for: Effectiveness of an Internet- and App-Based Intervention for College Students With Elevated Stress: Randomized Controlled Trial
Source: J Med Internet Res. 2018 Apr 23;20(4):e136. doi: 10.2196/jmir.9293 (PMC5938594; doi:10.2196/jmir.9293)
Supplement: Multimedia Appendix 4 [file jmir_v20i4e136_app4.pdf]

Participants' perceived usefulness, complexity and duration for each intervention module
(SD=standard deviation)

Ratings	Module	n	Mean	SD	%[a]
**Usefulness (1-5)**					
	Module 1	69	3.84	0.74	74
	Module 2	63	4.23	0.78	87
	Module 3	51	4.22	0.61	90
	Module 4	41	4.22	0.72	95
	Module 5	40	4.28	0.85	87
	Module 6	36	4.42	0.60	94
	Module 7	34	3.94	0.92	85
	Module 8	25	4.12	0.73	80
**Complexity (1-5)**					
	Module 1	68	2.12	0.84	7
	Module 2	63	2.67	0.97	24
	Module 3	51	2.41	0.88	12
	Module 4	41	2.07	0.96	45
	Module 5	40	2.63	1.03	20
	Module 6	36	3.14	1.04	44
	Module 7	34	1.91	0.75	3
	Module 8	25	1.76	0.78	0
**Duration (1-4)**					
	Module 1	68	2.26	0.75	6
	Module 2	63	2.65	0.84	16
	Module 3	51	2.92	0.74	24
	Module 4	41	2.34	0.66	2
	Module 5	40	2.80	0.85	20
	Module 6	36	3.03	0.74	28
	Module 7	33	2.06	0.97	9
	Module 8	25	1.56	0.65	0

[a]Percentage of ratings as:

- "useful" or "very useful" (Usefulness)
- "complex" or "very complex" (Complexity)
- "longer than 1½ hours" (Duration)
